# Anti Thymocyte Globulin-Based Treatment for Acquired Bone Marrow Failure in Adults

**DOI:** 10.3390/cells10112905

**Published:** 2021-10-27

**Authors:** Jennifer M.-L. Tjon, Saskia M. C. Langemeijer, Constantijn J. M. Halkes

**Affiliations:** 1 Department of Hematology, Leiden University Medical Centre, 2333 ZA Leiden, The Netherlands; J.M.L.Tjon@lumc.nl; 2 Department of Hematology, Radboudumc, 6500 HB Nijmegen, The Netherlands; saskia.langemeijer@radboudumc.nl

**Keywords:** acquired bone marrow failure, aplastic anemia, pure red cell aplasia, amegakaryocytic thrombocytopenia, pure white cell aplasia, myelodysplastic syndrome, anti-thymocyte globulin

## Abstract

Idiopathic acquired aplastic anemia can be successfully treated with Anti Thymocyte Globulin (ATG)-based immune suppressive therapy and is therefore considered a T cell-mediated auto immune disease. Based on this finding, several other forms of idiopathic acquired bone marrow failure are treated with ATG as well. For this review, we extensively searched the present literature for evidence that ATG can lead to enduring remissions in different forms of acquired multi- or single-lineage bone marrow failure. We conclude that ATG-based therapy can lead to an enduring hematopoietic response and increased overall survival (OS) in patients with acquired aplastic aplasia. In patients with hypocellular myelodysplastic syndrome, ATG can lead to a hematological improvement without changing the OS. ATG seems less effective in acquired single-lineage failure diseases like Pure Red Cell Aplasia, Amegakaryocytic Thrombocytopenia and Pure White Cell Aplasia, suggesting a different pathogenesis in these bone marrow failure states compared to aplastic anemia. T cell depletion is hypothesized to play an important role in the beneficial effect of ATG but, as ATG is a mixture of polyclonal antibodies binding to different antigens, other anti-inflammatory or immunomodulatory effects could play a role as well.

## 1. Introduction

Acquired bone marrow failure leading to a decreased production of one or more blood cell lineages is seen in adults in the context of a wide variety of diseases including hematological or non-hematological malignancies, (vitamin) deficiencies and external damage such as radiation therapy or toxic agents. In the absence of a clear cause for the impaired production of one or more blood cell lineages, the bone marrow failure is classified as idiopathic. In a part of these patients, the marrow failure seems to be caused by an auto-immune reaction as hematopoiesis can be improved by immune suppressive therapy [1].

Anti thymocyte globulin (ATG) is a mixture of polyclonal antibodies which is harvested from an animal (horse or rabbit) after immunization with cells from a human thymus or from a T-cell line (Jurkat) [2]. Although ATG contains antibodies that can bind to a variety of different antigens, partly depending on the type of cells used for immunization, the major therapeutic effect of ATG is attributed to the lytic effect on T cells. ATG is commonly used to prevent rejection of transplanted solid organs or to prevent graft versus host disease after allogeneic hematopoietic stem cell transplantation (HSCT). In addition, ATG has proven efficacy in the treatment of idiopathic acquired aplastic anemia (AA), a type of bone marrow failure characterized by a hypocellular bone marrow and bi- or trilineage cytopenia [3]. The observation of oligoclonal T cells and a decreased number of regulatory T cells in peripheral blood of AA patients at diagnosis in combination with recovery of hematopoiesis after ATG treatment has led to the hypothesis that AA is an autoreactive T cell-mediated disease [4]. It is not known whether the auto-immune reaction is targeted against the hematopoietic stem and progenitor cells (HSPCs) or the surrounding bone marrow niche. Furthermore, it has not been established whether T cells indeed are the causative immune effector cells that incite this auto-immune reaction resulting in bone marrow failure. 

In analogy to AA, it is hypothesized that other forms of idiopathic bone marrow failure including Pure Red Cell Aplasia (PRCA), Pure White Cell Aplasia (PWCA), Amegakaryocytic Thrombocytopenia (ATP) and some subtypes of Myelodysplastic Syndrome (MDS) are also auto-immune mediated. Based on the efficacy of ATG in AA, ATG has been applied for these forms of bone marrow failure as well. In this review we discuss the currently available evidence on the efficacy of ATG-based treatment for patients with acquired multi- or single lineage bone marrow failure. Currently, two different forms of ATG are available for this treatment: horse-derived ATGAM (Pfizer) and rabbit-derived Thymoglobulin (Sanofi). In this review we focus on these specific brands. Another horse-derived ATG has been used in the past as well (Lymphoglobulin), but this drug is no longer available. Due to the differences in dosing and composition of the horse ATG formulas, we chose to use the specific brand names in this review. If the specific brand name of a horse ATG was not mentioned in a paper, we use the term horse ATG and if the source of ATG (horse or rabbit) is not clear, we chose to use the term ATG.

## 2. ATG for Acquired Aplastic Anemia

AA is characterized by multi-lineage cytopenia and a hypocellular bone marrow. In a minority of the adult patients an underlying germline mutation predisposing to bone marrow failure is present and, in some cases, a viral infection was diagnosed prior to the development of the aplasia. However, most patients suffer from idiopathic AA. As mentioned before, idiopathic AA is hypothesized to be a T-cell-mediated disease. Therefore, one of the preferred first-line treatments is intensive immunosuppressive treatment consisting of ATG in combination with cyclosporin. Currently, two different forms of ATG are used as first-line treatment for idiopathic AA: horse-derived ATGAM (Pfizer) and rabbit-derived Thymoglobulin (Sanofi). For ATGAM, the results of several prospective trials investigating its role in first-line treatment have been published (Table 1). Champlin reported in 1983 the results of a randomized study in which 20 mg per kg bodyweight ATGAM was given for 8 days [5]. Eleven out of seventeen patients receiving ATGAM showed a hematological response (improvement of blood counts) in the 3 months after this treatment, compared to none of the patients in the control arm, receiving only transfusions as supportive treatment. It was hypothesized that ATG eliminated auto-reactive T cells and that the remaining hematological progenitor cells needed time to proliferate before recovery of peripheral blood cell count could be seen. Based on a study showing that the combination of Lymphoglobulin (a horse-derived ATG which is no longer available) and CyclosporinA (CsA) was superior to Lymphoglobulin alone [6], CsA was added to ATGAM for the treatment of AA. This combination resulted in stable response rates around 60% at six months after start of the treatment [7,8,9,10], see Table 1.

The addition of the thrombopoietin receptor agonist (Tpo-RA) Eltrombopag to the treatment with ATGAM and CsA in patients with acquired AA increased the overall response at six months to 87% [10]. The mechanism of action of Eltrombopag is presumed to be stimulation of the TPO receptor c-Mpl that is present on early progenitor cells resulting in expansion of these progenitors. 

There is some debate whether the results of first-line treatment with rabbit-derived Thymoglobulin are equivalent to the results of horse-derived ATGAM in acquired AA. The only randomized clinical trial comparing both drugs was set up to show a superior response rate of Thymoglobulin because Thymoglobulin leads to deeper and longer T cell depletion compared to ATGAM. As recovery of hematopoiesis is attributed to the elimination of lymphocytes that are autoreactive against HSPCs or their niche, Thymoglobulin was hypothesized to result in higher response rates. However, the overall response rate (ORR) at 6 months and overall survival (OS) 3 years after ATGAM was significantly better than after Thymoglobulin (68% versus 37% and 96% versus 76%, respectively) [9]. Other prospective studies showed response rates ranging from 40 to 64% at 6 months after Thymoglobulin [11,12,13] (Table 2) and it has been suggested that more responses occur at a later time point after Thymoglobulin. 

The difference in response rates between the two types of ATG is not fully understood, but could point to difference in lymphocyte types depleted between the ATG types. 

Based on the studies described above, European guidelines recommend horse-derived ATGAM in combination with CsA as first-line treatment for patients with idiopathic AA that are ineligible for first-line HSCT. The addition of Eltrombopag to first-line treatment with ATGAM and CsA is under investigation in a randomized trial (EBMT RACE trial). 

## 3. ATG for Acquired Pure Red Cell Aplasia

PRCA is characterized by a severe normocytic anemia, reticulocytopenia, and absence of erythroblasts in an otherwise normal bone marrow [14]. In children with PRCA, the congenital form (Diamond-Blackfan Anemia) should be excluded. In many patients with acquired PRCA, a concomitant disease is found. Parvovirus B19 can cause PRCA by directly infecting human erythroid progenitor cells through the red cell surface P antigen. The use of recombinant human erythropoietin (EPO) can lead to PRCA by the formation of autoantibodies to endogenous EPO [14]. PRCA can also evolve in the context of T-Large Granular Lymphocyte Leukemia (T-LGL) and there is an association between the presence of a thymoma and PRCA [15,16,17]. If no concomitant treatable disease has been found, acquired PRCA is termed idiopathic. Both idiopathic PRCA and the majority of PRCA cases associated with a concomitant disease are presumed to be caused by an auto-immune mechanism which directly interrupts the erythroid differentiation in the bone marrow.

Immunosuppressive therapy with corticosteroids, CsA or Cyclophosphamide leads to a hematologic recovery in the majority of patients [18]. Response to immunosuppression can occur both in patients with idiopathic PRCA and in patients with PRCA due to a concomitant disease. If a patient is refractory to first-line treatment with corticosteroids, CsA or Cyclophosphamide, ATG is suggested as a possible second- or third-line treatment. Table 3. shows the available evidence for efficacy of ATG in PRCA [19,20,21,22,23,24,25]. 

The majority of patients received horse-derived ATG. In the largest cohort of patients with acquired PRCA treated with ATGAM, four out of seven adult patients had hematological recovery of whom two appeared to have secondary PRCA (one had a chronic EBV reactivation and one suffered B-CLL). The PRCA patients who were reported to respond in the papers included in Table 3 often did not fulfill the diagnostic criteria for idiopathic PRCA as they suffered a concomitant hematological disease or had more than one lineage cytopenia (Table 3). No long-term follow-up is reported for the responding patients. 

Based on these data, the ORR for horse-derived ATG in a PRCA seems inferior compared to the outcome of horse-derived ATG based treatment in patients with AA. 

## 4. ATG for Acquired Amegakaryocytic Thrombocytopenia

Acquired Amegakaryocytic Thrombocytopenia (ATP) is characterized by a severe thrombocytopenia and the absence of megakaryocytes in an otherwise normal bone marrow. It can be seen in the context of diseases like systemic lupus erythematosus and infections. Both cytotoxic antibodies and T-lymphocytes were detected in patients with idiopathic ATP. Treatment with immunosuppressive drugs like corticosteroids, CsA or Cyclophosphamide are effective in part of the patients, but the ORR to these agents seem to be lower compared to the response rates in acquired PRCA [26].

Table 4 shows the published evidence for efficacy of ATG in acquired ATP [27,28,29,30,31,32]. Nine out of ten reported patients showed an enduring increase in platelet numbers after ATG treatment. Some of these patients had anemia and/or leukopenia at diagnosis and could be argued to have acquired AA, thereby explaining the response to horse-derived ATG. In the majority of the patients an increase in platelet number was already seen within 2 weeks after ATG infusion. Based on these data, horse-derived ATG can effectively lead to an increase in platelet number in acquired ATP. However, the time to response differs from the time to response in acquired AA, which could point to a difference in pathogenesis between ATP and AA. 

## 5. ATG for Acquired Pure White Cell Aplasia

In PWCA a selective hematopoietic failure of both the granulocyte and monocytic lineage is found with normal maturation of erythrocyte and megakaryocytic lineages. Associations have been described between PWCA and several malignancies such as thymoma, B-CLL or T-LGL, auto-immune disorders or the use of specific drugs [33,34,35]. Some case reports describe efficacy of treatment with intravenous immunoglobulins or Rituximab, suggesting a role for auto-antibodies in some patients [36]. We did not find reports on the efficacy of ATG-based therapy in idiopathic PWCA. 

## 6. ATG for Myelodysplastic Syndromes

Myelodysplastic syndromes (MDS) are a group of clonal hematopoietic stem cell disorders characterized by an ineffective hematopoiesis resulting in peripheral blood cytopenias and abnormal cellular maturation. Patients with MDS are at risk for progression to acute myeloid leukemia. There is great heterogeneity in both the extent of cytopenia and bone marrow cellularity. Therefore, patients can present with single- or multilineage disease and bone marrow with cellularity varying from hypocellular to hypercellular. Based on the number of lineages involved, blast count and cytogenetic abnormalities survival can be predicted (International Prognostic Scoring System (IPSS)) and patients can be classified into “lower risk” and “higher risk” MDS. This classification reflects the risk of disease progression, especially the risk for leukemic transformation. 

MDS arises from mutations in hematopoietic stem cells, that in the majority of patients originates from spontaneous mutations in the setting of aging. Disrupted inflammatory signals from the bone marrow niche may facilitate the occurrence and expansion of these mutated stem cells [37]. In a minority of patients, MDS is caused by cytotoxic chemotherapy, radiation, environmental factors or inherited genetic abnormalities (e.g., Fanconi anemia, dyskeratosis congenita, Schwachman-diamond syndrome). In addition, several cohort studies indicated an increased risk of MDS in patients with a history of autoimmune disorders, suggesting that chronic immune stimulation can act as a trigger for MDS development [38]. 

The treatment is aimed at improving cytopenia, reduction in transfusion dependency and delaying leukemic transformation. Allogeneic stem cell transplantation is the only curative option, but not feasible in most patients. Other treatment modalities that may improve cytopenia are transfusions, growth factors, hypomethylating agents and lenalidomide. The IPSS-R risk score and patient specific characteristics aid in the choice of a specific type of treatment. The preferred treatment of choice in higher risk MDS is an allogeneic stem cell transplantation or (in unfit patients/no candidate for SCT) treatment with hypomethylating agents, whereas in lower risk MDS a combination of transfusion and growth factors may be sufficient for hematologic improvement. Based on the possibility that MDS development is (in part) immune mediated, it was hypothesized that treatment with ATG, similar to the treatment of AA, could lead to hematologic recovery in MDS as well. 

In a phase II prospective study, 61 patients with MDS were treated with ATGAM 40 mg/day for 4 days intravenously (Table 5) [39]. The patients in this study all had a ECOG performance status of two or less and were dependent on erythrocyte transfusion with or without concurrent neutropenia or thrombocytopenia. To minimize the effect of previous treatments, patients had to discontinue all other treatments capable of stimulating bone marrow (e.g., growth factors, cyclosporin, steroids) at least 1 month before entering the study. Patients with 20% or more bone marrow blasts were excluded. Twenty-one of 61 patients (34%) became transfusion independent within 8 months of ATG treatment, with transfusion independence as the main criterion for hematological response. In addition, all 21 responders had neutropenia with or without thrombocytopenia that normalized after ATG treatment. Five-year overall survival rate was 64% for all 61 patients. It is important to note that from the 21 responders, only 1 died before the end of the study whereas 22 from the 40 non-responding patients died. Factors predictive of response to ATG were younger age and bone marrow hypocellularity [39]. In a prospective multicenter trial, 88 patients with low to intermediate risk were randomly assigned to either 5 days of 15 mg/kg horse ATG (Lymphoglobulin) in combination with CsA for 180 days or to best supportive care consisting of transfusion support and growth factors. At 6 months, 29% of the patients in the intervention arm achieved hematological response (transfusion independence, with improvement of peripheral blood counts). The two-year overall survival rate in the intervention arm was 49%, which did not differ significantly from the overall survival in the best supportive care arm. This study also showed that response was significantly associated with bone marrow hypocellularity [40]. Finally, a retrospective cohort study of 207 patients with low risk MDS treated with various types of immunosuppressive therapy showed a significant association between achievement of transfusion independence and IST consisting of horse-ATG in combination with cyclosporin and bone marrow hypocellularity [41].

In conclusions, these studies show that IST consisting of ATG with or without cyclosporin can lead to transfusion independency in MDS, in particular hypocellular MDS.

## 7. Discussion

Acquired bone marrow failure syndromes encompass a heterogeneous group of disorders characterized by single or multi lineage cytopenia. In a subset of patients, immunosuppressive treatment can lead to recovery of hematopoiesis, strongly suggesting an autoimmune mediated origin of bone marrow failure. The most successful among immunosuppressive treatments is horse-derived ATG-based treatment for acquired AA [42]. Based on the success of horse-derived ATG in AA, this treatment has also been used in other types of acquired bone marrow failure. In this review we set out to evaluate the available evidence for the use of ATG as treatment for acquired bone marrow failure.

In general, horse-derived ATG with or without CsA has been mostly studied in the multi-lineage bone marrow failure syndromes AA and MDS. In the two available randomized trials in patients with acquired AA, ATGAM led to an improvement of blood counts and an improved OS compared to best available supportive care or to Thymoglobulin, a rabbit-derived ATG. In patients with MDS, horse-derived ATG (Lymphoglobulin or ATGAM) in combination with CsA improved blood counts but did not lead to an increased OS. Furthermore, response to horse-derived ATG with or without CsA in MDS patients was mainly associated with bone marrow hypocellularity. Some case reports suggest efficacy of ATG in acquired single lineage bone marrow failures like ATP and PRCA, but data based on well-designed prospective studies are lacking, which is not surprising in the light of the low incidence and heterogeneity of these diseases. A clear recommendation regarding the use of ATG in single lineage bone marrow failure is therefore not possible.

Recovery of hematopoiesis after treatment with ATG strongly suggests an auto-immune pathogenesis in acquired bone marrow failure. However, the origin of the effector cells involved and the exact target of this auto-immune reaction are not clear. In AA, oligoclonal expansion of T cells has been associated with the (re) occurrence of bone marrow failure. A widely accepted view is that these oligoclonal T cells are autoreactive T cells that target HSPCs resulting in bone marrow failure. It is hypothesized that ATG results in depletion of autoreactive lymphocytes that have initiated an immune cascade resulting in an ongoing effector phase. This could explain why despite the apparent quick recovery of lymphocytes after ATG treatment (within weeks) regeneration of bone marrow function takes several months. However, lymphocyte depletion in general seems not the only factor leading to recovery of hematopoiesis. This view is supported by the fact that rabbit-derived Thymoglobulin, which leads to a more potent and prolonged T cell depletion when compared to horse-derived ATGAM, is less effective in the treatment of AA. It has been suggested that a disturbed balance between effector and regulatory T cells contributes to the development of AA and that treatment with ATGAM restores this balance. However, ATG contains a wide variety of antibodies binding to different targets and cells such as endothelial cells, B cells and antigen presenting cells. It can be argued, therefore, that the therapeutic effect of ATGAM goes beyond lymphocyte depletion and that binding of antibodies present in the ATGAM mixture to other cells than T lymphocytes contribute to the beneficial effect in bone marrow failure as well.

A randomized study showed that treatment with horse-derived ATG could lead to hematological improvement in patients with MDS, in particular hypocellular MDS. There was, however, no significant difference in the overall survival between the intervention arm and the control group. Furthermore, the leukemic transformation rate was equal between the control and intervention group. These findings indicate that ATG can lead to temporal improvement of blood counts in hypoplastic MDS without changing the natural course of this disease. The distinction between acquired AA and hypoplastic MDS can be challenging as dysplastic features can be difficult to visualize in a hypocellular bone marrow. Part of the patients who have been treated with ATG for AA eventually develop AML or MDS. This could be caused by the outgrowth of mutated HSPCs already present at the time of ATG treatment. Another explanation is that these mutations occur under pressure in the limited pool of dividing hematopoietic cells after treatment with ATG. Currently available techniques like targeted sequencing can reveal the presence of MDS associated mutations in bone marrow at diagnosis and during follow up after ATG treatment. The recently closed randomized EBMT Race study that studies the addition of Eltrombopag to the standard first-line treatment with ATGAM and CsA in patients with acquired AA will shed light on the presence and development of these mutations before and after treatment.

In conclusion, although the exact working mechanism of ATG is unknown, ATG based immune suppressive treatment can lead to enduring responses in patients with acquired aplastic anemia. In patients with hypoplastic MDS ATG can lead to an improvement of blood counts but does not seem to change the natural course of the disease. For acquired single line bone marrow failures such as PRCA, ATP or PWCA, there is limited or no evidence that ATG leads to enduring responses.

## Figures and Tables

**Table 1:** Published prospective studies on ATGAM as first line therapy in acquired AA. d: days, ORR: overall response rate, OS: overall survival.

Study	Treatment	Outcome
Intervention arm in a prospective, randomized study 1979–1981, *n* = 17 [5]	ATGAM 20 mg/kg/day, 10 d	3m: ORR 11/17
Prospective single arm study 1991–1999, *n* = 122 [7]	ATGAM 40 mg/kg/day, 4 d Cyclosporin 6 months	6m ORR 61% OS 55% at 7 years
Control arm in a prospective, randomized study 2003–2005, *n* = 42 [8]	ATGAM 40 mg/kg/day, 4 d Cyclosporin 6 months	6m ORR 62% OS 90% at 3 years
Control arm in a prospective, randomized study 2005–2010, *n* = 60 [9]	ATGAM 40 mg/kg/day, 4 d Cyclosporin 6 months	6m ORR 68% OS 96% at 3 years
Prospective cohort study 2012–2015, *n* = 92 [10]	ATGAM 40 mg/kg/day, 4 d Cyclosporin 6 months Eltrombopag 1 d 150 mg	6m ORR 87% OS 97% at 2 years

**Table 2:** Published prospective studies on Thymoglobuline as first line therapy in acquired AA. d: days, ORR: overall response rate, OS: overall survival.

Study	Treatment	Outcome
Intervention arm in a prospective, randomized study 2005–2010, *n* = 60 [9]	Thymoglobulin 3.5 mg/kg/day, 5 d Cyclosporin 6 months	6m ORR 37% OS 76% at 3 years
Prospective single arm study 2005–2009, *n* = 20 [11]	Thymoglobulin 2.5 to 4.0 mg/kg/day, 5 d Cyclosporin	6m ORR 45%
Prospective single arm study 2008–2010, *n* = 35 [12]	Thymoglobulin 3.75 mg/kg/day, 5 d Cyclosporin 6 months	6m ORR 40% OS 68% at 2 years
Prospective single arm study, *n* = 24 [13]	Thymoglobulin 3.5 mg/kg/day, 5 d Cyclosporin 6 months	Cumulative ORR 64% OS 70% at 3 years

**Table 3:** Published studies on ATG as treatment in acquired PRCA. d: days, ORR: overall response rate, CR: complete remission.

Study	Treatment	Outcome	Remark
Case serie 1986, *n* = 7 adult [19]	ATGAM 15 mg/kg/d, 10 d	ORR 4/7	Of responding patients: 1 had B-CLL, 1 had chronic EBV infection
Case serie 1984 *n* = 3 adult [20]	Horse ATG, no details on dosing	ORR 0/3	
Case serie 1985 *n* = 2 [21]	ATG, no details on dosing	ORR 2/2	Response starting at 2–4 days
Case serie 2001 *n* = 2 [22]	Horse ATG 15 mg/kg/d, 5 d Horse ATG 20 mg/kg/d, 8 d	Deceased Response at week 4	Follow-up 8 weeks
Case 1988 [23]	Horse ATG 50 mg/kg/day, 5 days	Responded with duration >2 year	Decreased platelet number and WBC at diagnosis
Case 1988 [24]	Horse ATG 40 mg/d, 10 d	Responded	B-CLL at diagnosis
Cohort 2018 [25]	ATG, no details on dosing	3/10 (1 CR)	2/3 responding patients had T-LGL associated PRCA

**Table 4:** Published studies on ATG as treatment in acquired amegakaryocytic thrombocytopenia. d: days, ORR: overall response rate, CR: complete remission.

Study	Treatment	Outcome	Remark
Case serie 2008 *n* = 4 [27]	ATG 3,5 mg/kg/d, 4 d Cyclosporin 6 months	ORR 3/4, CR 28–178 days	CR after 28–178 days, lasting 16–60 months
Case serie 1999 *n* = 2 [28]	ATG 37.5 mg/kg/d, 8 d Cyclosporin 6 months	ORR 2/2	Case 1 response after 2 weeks, relapse after discontinuation of CsA Case 2 response within 2 weeks
Case 1986 [29]	ATG 40 mg/kg/d, 4 d	Responded 7 weeks after ATG	Pancytopenia at diagnosis
Case 2001 [30]	ATG 15/mg/kg/d 5 d	Responded at 10 days after ATG	PRCA and AMT
Case 2004 [31]	ATG 40 mg/kg/d, 4 d	Responded within a week	Eosinophilic Fasciitis
Case 1985 [32]	Lymphoglobulin 150 mg/kg total	Responded within a week	

**Table 5:** Published studies on ATG as treatment in MDS. d: days, y: year, ORR: overall response rate, CR: complete remission.

Study	Patients	Treatment	Outcome	Remark
Phase II, single arm prospective study 1994–1998 [39]	*n* = 61 Excl: CMML, >20% BM blasts	ATGAM 40 mg/kg iv 4 d	ORR at 8 months: 21/61 = 34% 5y OS: 64% (CI 52–76)	BM hypocellularity almost significant factor predicting response
Phase III multicenter RCT 2000–2006 [40]	*n* = 88	Lymphoglobulin 15 mg/kg 5 d+ oral CsA 180 d *n* = 45 Best supportive care(transfusion, growth factors) *n* = 43	OR 6 months intervention arm: 13/45 =29% 2y OS intervention arm: 49% (CI 31–66) 2y OS BSC arm: 59% (CI 38–75)	Patients with BM hypocellularity (*n* = 9) 50 % RR to ATG + CsA
Retrospective cohort study multicenter (15) 2006–2016 [41]	*n* = 207 Low risk MDS Incl: treated with IST Excl: monotherapy prednisone	Rabbit or horse ATG, CsA, tacrolimus, alemtuzumab and combinations of these NB: ATG based combinations 76%	125 pts with response data ORR 48.8% Median OS in 207 pts: 47.4 months	Hypocellular BM and horse ATG + CsA were significantly associated with achievement of transfusion independence

## Data Availability

Not applicable.
